# Parainfluenza Virus 5 as Possible Cause of Severe Respiratory Disease in Calves, China

**DOI:** 10.3201/eid2112.141111

**Published:** 2015-12

**Authors:** Ye Liu, Nan Li, Shoufeng Zhang, Fei Zhang, Hai Lian, Rongliang Hu

**Affiliations:** Laboratory of Epidemiology and Key Laboratory of Jilin Province for Zoonosis Prevention and Control, Academy of Military Medical Sciences, Changchun, China

**Keywords:** Parainfluenza virus 5, calves, paramyxovirus, viruses, China, zoonoses, severe respiratory disease

**To the Editor:** Parainfluenza virus 5 (PIV5), family *Paramyxoviridae,* genus *Rubulavirus,* was previously known as simian virus 5 because of its discovery in primary monkey kidney cells in 1954 (*1*). PIV5 was later isolated from various hosts, including humans, dogs, pigs, cats, and rodents. The neutralizing antibody for PIV5 is detectable in symptomatic and asymptomatic humans; thus, its association with human disease remains controversial (*2*). In addition, previous studies have not documented illness in infected animals, except kennel cough in dogs (*1*,*3*). Isolation of PIV5 from cattle has not previously been reported.

Since 2012, an infectious respiratory disease has been prevalent in weaning calves (≈10 d to 2 mo of age) in Baicheng City, Jilin Province, China. Initial clinical signs included secretion of clear nasal mucus, anorexia, sluggishness, and loss of bodyweight. After 10–20 d, ≈10% of the sick calves died of dyspnea and interstitial pneumonia. Farmers observed that 80%–90% of calves in the affected farms demonstrated clinical signs, but most recovered. All attempts of local veterinarians to treat the animals with various chemical compounds and antimicrobial drugs failed. The disease persists throughout the year but occurs mainly during spring (from February through March), which has resulted in substantial economic losses in the cattle industry.

To identify the causative agent of the disease, we tested 15 lung samples from calves that had died and 10 lung samples from healthy calves that were slaughtered for serum products (all from 1 farm). The samples were homogenized in phosphate-buffered saline and analyzed by using electron microscopy. Paramyxovirus-like particles were identified in the tissues of sick calves. Reverse transcription PCR with the generic primers for the paramyxovirus polymerase gene was performed (*4*).

Of the 25 calf specimens, the 15 samples from the sick calves were positive by reverse transcription PCR, and amplicons of the expected size were obtained and sequenced. The generated sequences were closely related to the PIV5 sequences available from GenBank, particularly to sequences of the recently identified KNU-11 and SER viruses in pigs (*5*,*6*).

The suspensions of lung tissue from sick calves were purified by centrifugation at 12,000 × *g* for 5 min, and 0.2 mL of the supernatant was added to a Vero cell monolayer in a 25-cm^2^ cell culture flask (EasyFlasks; Thermo Fisher Scientific, Odense, Denmark). After virus adsorption for 1 h at 37°C, the cell monolayer was rinsed with phosphate-buffered saline (pH 7.4) and then incubated in Dulbecco minimal essential medium/2% newborn calf serum at 37°C in a 5% CO_2_- humidified incubator. The infected cells were serially passaged every 3 days at 37°C and detected by using monoclonal antibody against SV5 (AbD Serotec; Bio-Rad, Kidlington, UK) by indirect fluorescent antibody test (*7*). A PIV5 strain was isolated in the cell culture and designated PIV5-BC14 (BC14 stands for Baicheng City 2014).

For amplification and analysis of the full-length viral genome, 13 pairs of primers covering overlapping fragments of the genome were designed on the basis of the sequence of the PIV5 isolate KNU-11 (*8*). The 3′ and 5′ termini of the genome were resolved by using the 3′ and 5′ Full RACE Kit (TaKaRa Biotechnology Co., Ltd., Dalian, China). The PIV5-BC14 genome (GenBank accession no. KM067467) was 15,246 nt with coding and untranslated regions at the same positions as in other PIV5 isolates (*9*). However, comparison of this genome with 15 available full-length genomes of PIVs revealed that 18 nt substitutions, resulting in 9 aa changes, are found only in PIV5-BC14. Among these 9 aa changes, 5 (at positions 303, 634, 1054, 1722, and 1773) are present in an RNA-dependent RNA polymerase protein, 2 in a phosphoprotein (at positions 43 and 332), 1 in a nucleoprotein (at position 75), and 1 in a hemagglutinin–neuraminidase (at position 322).

The highest nucleotide identity (99.72%/99.52%) was observed between PIV5-BC14 and porcine PIV, particularly with the SER virus isolate. This observation was confirmed after construction of a phylogenetic tree based on the 15 available nucleotide sequences of the full-length genomes (Figure). The analysis was carried out by using the maximum-likelihood method in MEGA 5.0 (*10*), and the reliability of tree topology was evaluated through bootstrapping with 1,000 replicates.

**Figure:**
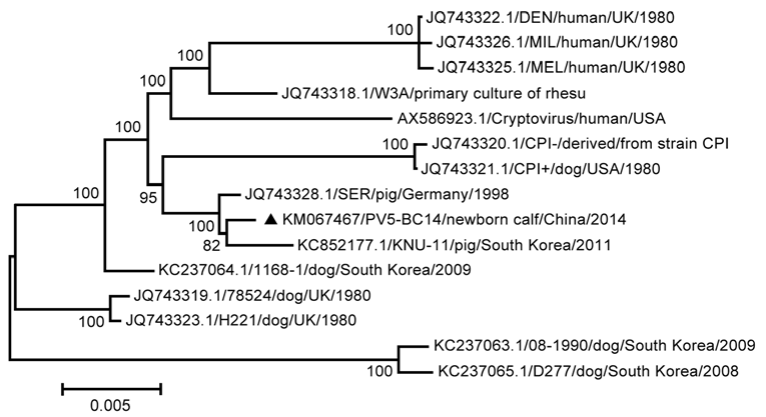
Maximum-likelihood phylogenetic tree based on the complete genome sequences of parainfluenza virus 5 (PIV5). The black triangle indicates isolate PIV5-BC14 (Baicheng City 2014). Scale bar indicates nucleotide substitutions per site.

Although the pathogenic role of PIV5 infections in cattle remains unknown, no PIV5 RNA was found in any apparently healthy cattle from the same farm. This result suggests a strong relationship between the identified virus and the disease.
